# The genetic parameters of feed efficiency and its component traits in the turkey (*Meleagris gallopavo)*

**DOI:** 10.1186/1297-9686-44-2

**Published:** 2012-01-23

**Authors:** Lindsay A Case, Benjamin J Wood, Stephen P Miller

**Affiliations:** 1 Department of Animal and Poultry Science, University of Guelph, Guelph, ON, Canada; 2 Hybrid Turkeys, Suite C, 650 Riverbend Drive, Kitchener, ON, Canada

## Abstract

Residual feed intake (RFI) and feed conversion ratio (FCR) can be incorporated into a breeding program as traits to select for feed efficiency. Alternatively, the direct measures used to calculate RFI and FCR can be analyzed to determine the underlying variation in the traits that impact overall efficiency. These constituent traits can then be appropriately weighted in an index to achieve genetic gain. To investigate feed efficiency in the turkey, feed intake and weight gain were measured on male primary breeder line turkeys housed in individual feeding cages from 15 to 19 weeks of age. The FCR and RFI showed moderate heritability values of 0.16 and 0.21, respectively. Feed intake, body weight, and weight gain were also moderately heritable (0.25, 0.35, and 0.18, respectively). Weight gain was negatively correlated to feed conversion ratio and was not genetically correlated to RFI. Body weight had a small and positive genetic correlation to RFI (0.09) and FCR (0.12). Feed intake was positively genetically correlated to RFI (0.62); however, there was no genetic correlation between feed intake and FCR. These estimates of heritability and the genetic correlations can be used in the development of an index to improve feed efficiency and reduce the cost of production.

## Background

Feed represents two thirds of the total costs of poultry production and feed requirements are an important consideration in the turkey industry. Furthermore, as genetic progress is made in body weight traits, feed consumption could be expected to increase since larger birds require more feed. Consequently, improving feed efficiency by identifying animals that require the same amount of feed as their contemporaries but have higher body weight or weight gain is valuable in the animal production industry. This is feasible in a breeding program, and genetic selection combined with management, has improved feed conversion ratio (FCR) in the turkey by approximately 20% between 1966 and 2003 [1].

Feed efficiency is often assessed as either FCR or residual feed intake (RFI) [2]. The ratio of feed intake to weight gain, or FCR, provides an indication of a bird's ability to convert feed to body weight, however selection based on a ratio is not ideal [3]. The RFI trait attempts to isolate a measure of biological efficiency independent of production, which can include weight gain or carcass yield, and is estimated as the difference between actual feed intake and a predicted feed intake based on body weight and production [2]. The RFI and FCR traits have moderate heritability values in broilers [4,5]; however published parameters for feed efficiency traits including RFI in the turkey have not been reported.

Reliable genetic parameters are essential for selection index design and to determine the correlated response to selection that will occur in other traits in a selection index. As a result, accurate estimates of genetic parameters enable turkey breeders to determine the impact that selection for feed efficiency will have on a breeding program. The objective of the present study was to determine the heritability of RFI and FCR, as well as their component traits in the turkey and to determine genetic correlations between the traits.

## Methods

### Population studied and management

Data collected on toms from a primary breeder turkey sire line (n = 16 412) over a 10-year period were used and breeding objectives emphasized commercial performance traits including growth, feed efficiency, and meat yield. Pedigree information extended back a minimum of five generations and included 28 464 relatives of the birds with records. Rearing until 14 weeks of age was under a standard commercial production environment and feeding regime, which involved group housing and a feeding program using shared feeders distributed on the basis of bird density. At 14 weeks of age, toms were placed in individual cages (0.60 m wide, 0.85 m long, 0.82 m high) to acclimatize and the turkeys remained in the same cages throughout the feeding trial. The feeding trial was conducted from approximately 15 to 19 weeks of age, since this corresponds to the rapid growth phase and is the standard time period used to assess feed efficiency in the commercial breeding program. Body weight was measured at the start of the trial (15 weeks of age) and at the end of the trial (19 weeks of age) and feed intake was recorded. During this period toms were fed a standard commercial diet [6] and had *ad libitum *access to feed and water from individual feeders and shared drinkers within each cage.

### Data analysis

Average daily gain (ADG) was calculated as:

$$ADG=\frac{\text{weight at end of trial (kg) - weight at start of trial (kg)}}{\text{days on trial}}$$

and metabolic mid-weight (MMW) was calculated as:

$$MMW={\left(\frac{\text{weight at end of trial (kg) + weight at start of trial (kg)}}{2}\right)}^{0.75}.$$

Feed conversion ratio (FCR) was calculated as feed intake divided by weight gain. Means and standard deviations for the traits measured are shown in Table 1. Expected feed intake was calculated using two different regressions (M1 and M2) as:

**Table 1:** Means and standard deviations of measured traits

Traits^1^	Units	Mean	Standard deviation
**FCR**	kg/kg	2.96	0.46
**FI**	kg	18.56	3.50
**BW**	kg	13.58	1.46
**MMW**	kg	0.42	0.08
**ADG**	kg	0.23	0.06

^1 ^Feed conversion ratio (FCR), cumulative feed intake (FI), body weight at 15 weeks (BW) and weight gain (ADG) as recorded during a 4 week feeding trial

$$\text{M}1:F1=\mu +b_{1}MMW+b_{2}WG+hatch+e$$

$$\text{M2}:F1=\mu +b_{3}BW+b_{2}WG+hatch+e$$

where *FI *represents feed intake, μ is the intercept, *b_1_, b_2_*, and *b_3 _*represent the regression coefficients on metabolic mid-weight, weight gain (WG), and body weight (BW) at the start of the trial, respectively. A fixed contemporary group effect (*hatch*) adjusted for the common environment effect that influenced a group of birds hatched on the same date and managed in the same contemporary group. The residual effect was represented by *e*. The R-squared value of M2 was higher than that of M1 and consequently coefficients from M2 were used to calculate RFI as:

$$RFI=FI-\left(\hat{\mu }+{\hat{b}}_{1}BW+{\hat{b}}_{2}WG\right)$$

Data exceeding three standard deviations from the mean were removed as outliers for each trait and the remaining 15 831 individuals with records for all traits were used for the genetic analysis.

### Genetic analysis

Heritabilities, phenotypic, and genetic correlations were estimated using ASReml [7]. The model for all traits was:

Trait=hatch+animal+e

where *Trait *represents RFI, FCR, FI, BW, or WG, *hatch *was the same fixed contemporary group effect used in the M1 and M2 RFI models, *animal *represented the random additive genetic effect, and *e *was the residual random effect. The random effects were assumed to be normally distributed with a mean of zero and a (co)variance structure equal to:

$$V\left(\begin{array}{c} a\\ e \end{array}\right)=\left(\begin{array}{cc} \text{A}\sigma_{a}^{2} & 0\\ 0 & \text{I}\sigma_{e}^{2} \end{array}\right)$$

where **A **represents the additive genetic relationship matrix and **I **is an identity matrix. Phenotypic and genetic correlations were estimated pair-wise in bivariate models and the reported heritabilities were averages across the bivariate models.

## Results

Means and standard deviations of the measured traits are shown in Table 1. The R-squared value of M2 (0.79) was higher than that of M1 (0.75) and as a result, body weight at the start of the trial explained a larger proportion of the variation in feed intake than metabolic mid-weight. For this reason, the regression coefficients from M2 were used to calculate RFI. The R-squared value indicates that RFI may account for up to 21% of the remaining variation in feed intake after adjusting for body weight, weight gain, and hatch.

Heritabilities, phenotypic, and genetic correlations are shown in Table 2. All traits showed a moderate heritability (0.16-0.35). The phenotypic and genetic correlations between feed intake, body weight, and ADG were all positive and within the moderate to high range with genetic correlations ranging from 0.28-0.67. There were also high positive phenotype and genetic correlations between RFI and FCR. The genetic correlation between feed intake and RFI (0.62) was positive but the correlation between feed intake and FCR was approximately zero. The ADG trait was negatively correlated to FCR (-0.67) but was not genetically correlated to RFI (-0.04 ± 0.07). The BW trait was not phenotypically correlated to RFI, as was expected, but there was a weak positive genetic correlation. The phenotypic (0.10) and genetic (0.12) correlations between BW and FCR were low and positive.

**Table 2:** Heritabilities (on diagonal), phenotypic (above diagonal) and genetic (below diagonal) correlations, plus or minus standard errors

Traits^1^	RFI	FCR	FI	BW	ADG
**RFI**	**0.21 ± 0.02**	0.49 ± 0.01	0.66 ± 0.01	0.00 ± 0.01	0.06 ± 0.01
**FCR**	0.65 ± 0.05	**0.16+0.02**	-0.13 ± 0.01	0.10 ± 0.01	-0.74 ± 0.00
**FI**	0.62 ± 0.04	0.06 ± 0.07	**0.25 ± 0.02**	0.41 ± 0.01	0.55 ± 0.01
**BW**	0.09 ± 0.06	0.12 ± 0.07	0.67 ± 0.04	**0.35 ± 0.02**	0.17 ± 0.01
**ADG**	-0.04 ± 0.07	-0.67 ± 0.04	0.28 ± 0.06	0.41 ± 0.06	**0.18 ± 0.02**

^1^Resdiual feed intake (RFI), feed conversion ratio (FCR), cumulative feed intake (FI), body weight at 15 weeks (BW) and weight gain (ADG) as recorded during a 4 week feeding trial

## Discussion

The feed efficiency traits (FCR and RFI) that were analysed could both be considered for a selection index and the magnitude of heritability indicated that selection could effectively improve each trait. The estimated heritability of BW agrees with previous estimates in the turkey [8] and the heritabilities of RFI and feed intake agree with reported moderate estimates in broiler chickens [4,9]. The heritability of FCR was intermediate between results in these studies (0.12 - 0.16) and lower than values from a recent study on broilers, which ranged from 0.41 - 0.49 [5].

Genetically, the FCR trait was negatively correlated to ADG (-0.67) and this relationship between FCR and weight gain also exists in the chicken and beef cattle with genetic correlations of -0.50 and -0.52, respectively [4,10]. These correlations are expected because of the relationship between feed conversion ratio and its component trait of ADG. The RFI trait was adjusted for body weight, and, as expected, the phenotypic and genetic correlations between RFI and body weight were low. Near-zero correlations between RFI and body weight at the start of a trial have also been estimated in meat-type chickens [4].

To account for the effect of body weight on maintenance energy requirements, RFI can be calculated by adjusting for either BW, as in the present study, or MMW. In a study on broilers, RFI was calculated using a regression on MMW in place of body weight and positive genetic correlations (0.29 - 0.49) between MMW and RFI were estimated [5]. Meat-type poultry have a very high and non-linear growth rate, and these results in broilers indicate that the mid-trial MMW measurement, which inherently includes the effect of growth throughout the trial, may confound the independence of RFI from this body weight trait. Consequently, body weight at the start of a trial may be a more beneficial trait to use, relative to MMW mid-trial, in the calculation of RFI in poultry to remove the genetic correlation between feed efficiency and body weight. The use of BW in the calculation of RFI would allow the analysis of the metabolic, nutritional, and physiological factors that contribute to RFI independently of the nutritional needs required to maintain body weight and to gain weight. As the investigation of feed efficiency in the turkey expands in the literature, trait parameters could also be reported for female birds. Evidence in broilers has shown that the relationship between efficiency and production traits change over the growing period [5], and as a result FCR and RFI should be evaluated at an earlier age in hens to evaluate both male and female turkeys during the rapid growth phase.

RFI showed a high genetic correlation to the feed conversion trait, which was expected based on results from other species [10,11]. The positive genetic correlation estimated between feed intake and RFI was favorable since increased efficiency was associated with a decrease in feed intake. Alternatively, the genetic correlation between feed intake and FCR was near zero indicating that improvement of one trait may not impact the other. This correlation was unexpected based on the inherent relationship between the traits and positive correlations in beef and swine, however a near-zero correlation has also been estimated in a study on broilers [4,10,11]. Studies in broilers have also shown that selection for FCR has a negligible impact on feed intake and a zero genetic correlation was estimated between feed intake and FCR in lines selected for feed efficiency [12]. Consequently, results suggest that RFI may be more favorably correlated to feed intake and more independent of performance traits than FCR.

Ideally, feed efficiency measures would be independent of production and this was shown for RFI. This indicates that the genetic regression between ADG and RFI was equal to the phenotypic regression, to produce a genetic independence between the traits [13], and selection for RFI would likely not have an impact on ADG. Additionally, the correlations between feed efficiency traits and traits associated with body composition such as breast meat yield were not considered here. These relationships should be considered prior to implementation into a breeding program as important genetic relationships with body conformation traits, like back fat, muscle area, and meat yield have been observed in other species [10,14].

Previous research demonstrated that a linear selection index can be more efficient than selection on a ratio trait like FCR [3,15]. A direct measure of feed efficiency can be omitted from an index, without decreasing the accuracy, if it is developed with appropriate weights on the component traits (feed intake, body weight, and growth) [13]. However, RFI may be advantageous because it can be considered independently, unlike feed intake, which is difficult to interpret as a standalone trait independent of growth rate and body weight. As a result, genetic improvement for efficiency can be accomplished by including a feed efficiency trait in a selection index or through its component traits, with the appropriate parameters as presented here.

## Conclusions

These results provide the required genetic parameters of feed efficiency in the turkey for consideration in the development of a breeding program. The heritabilities and genetic correlations can be used to calculate selection index weights. The moderate heritability of feed efficiency traits and the genetic correlations to feed intake, weight gain, and body weight indicate that selection can improve feed efficiency. The direct measures of FCR and RFI can be best utilized to study the traits that impact overall efficiency. A breeding program can then use this information to incorporate the efficiency constituent traits into an index to achieve genetic progress. This will, ultimately, reduce the relative cost of production and improve profitability for the turkey industry.

## Competing interests

The authors declare that they have no competing interests.

## Authors' contributions

All authors were involved in the conception of the study as well as study design. LC performed the data analysis, interpretation, and wrote the manuscript. BW was primarily responsible for critical revisions of the manuscript drafts. All authors read and approved the final manuscript.
